# The unique diagnostic and management challenge of a patient with concomitant anti-interferon-gamma autoantibody associated immunodeficiency syndrome, IgG4-related disease, and treatment refractory, disseminated mycobacterium avium complex infection

**DOI:** 10.1186/s13223-022-00722-x

**Published:** 2022-09-09

**Authors:** Spencer Boyle, Ashley Hagiya, Minh-Vu H. Nguyen, Howard Liebman, Jin Sol G. Lee

**Affiliations:** 1 Department of Internal Medicine, Keck School of Medicine of University of Southern California (USC), Lausanne, Switzerland; 2 Department of Clinical Pathology, Keck School of Medicine of University of Southern California (USC), Waltham, USA; 3 Department of Internal Medicine, Division of Infectious Diseases, University of California, Oxford, England; 4 Department of Internal Medicine, Jane Ann Nohl Division of Hematology and Center for the Study of Blood Diseases, Keck School of Medicine of University of Southern California (USC), Oxford, England; 5 Department of Internal Medicine, Section of Hospital Medicine, Division of Geriatric, Hospital, Palliative & General Internal Medicine at Keck School of Medicine of University of Southern California (USC), Hoboken, USA

**Keywords:** Anti-interferon-gamma autoantibody, Adult onset immunodeficiency, AOID, AOIS, IgG4, IgG4-related disease IgG4-RD, Disseminated MAC

## Abstract

**Background:**

Anti-interferon-gamma autoantibody-associated immunodeficiency syndrome is a rare and underrecognized adult onset immunodeficiency syndrome associated with severe opportunistic infections such as disseminated nontuberculous mycobacterium. Few cases have documented a relationship with IgG4-related disease. Concomitant diagnoses of these diseases present a diagnostic and management challenge.

**Case presentation:**

A 61 year old man of Southeast Asian descent with pulmonary mycobacterium avium complex infection presented to our hospital system with a new skin rash and worsening lymphadenopathy. He was eventually diagnosed with IgG4-related disease through excisional nodal biopsy. He was managed with immunosuppressive treatment with prednisone, rituximab and cyclophosphamide. He later re-presented with disseminated mycobacterium avium complex infiltration of his joints, bones and prostate. Original titers of anti-interferon-gamma autoantibodies were falsely negative due to being on immunosuppressive therapy for his IgG4-related disease. However, anti-interferon-gamma autoantibody titers were re-sent after immunosuppression was held and returned strongly positive.

**Conclusions:**

This case reviews diagnostic criteria and discusses management strategies with existing challenges in treating a patient with concomitant adult onset immunodeficiency syndrome, IgG4-related disease and a disseminated mycobacterial avium complex infection.

## Background

Anti-interferon-gamma autoantibody-associated immunodeficiency syndrome is an emerging adult onset immunodeficiency (AOID) disease. Acquired, neutralizing autoantibodies inhibit the downstream signaling of interferon (IFN)-gamma causing an increased susceptibility to intracellular pathogens leading to severe disseminated infections, especially nontuberculous mycobacterial (NTM) species [1]. First described in 2004, this disease predominantly affects patients of Southeast Asian descent and is characterized by reactive dermatoses and persistent, disseminated infections [2, 3].

Immunoglobulin G4-related disease (IgG4-RD) can be misdiagnosed given its clinical presentation can mimic other disease processes including infections, cancer, or other immune mediated conditions. As a result, the true prevalence of IgG4-RD is unknown. The reported incidence rate of IgG4-RD was estimated to be around 1 case for every 10,000 residents in Japan [4]. However, since the establishment of unified diagnostic criteria in 2011, we have seen a growing number of IgG4-RD cases reported internationally within the literature [4, 5].

Due to the novelty of both IgG4-RD and AOID, the natural history of these diseases is often described through case report and remain poorly understood. Thus, management continues to be a challenge.

Furthermore, a concomitant diagnosis of AOID and IgG4-RD has only been described twice in the literature [6, 7]. Treatment for AOID and IgG4-RD includes immunosuppression with steroids and steroid sparing biologic agents. Consequently, this presents a complex clinical challenge for a patient population at risk for infection. We highlight a novel case of a patient with IgG4-RD and AOID with recurrent, disseminated Mycobacterium avium complex (MAC) infection, posing a unique diagnostic and management challenge. In this case report, we review:

1. The diagnostic criteria of IgG4-RD and diagnosis of AOID
2. The pathophysiologic mechanisms of overlap between the two diseases
3. The literature on management of IgG4-RD and AOID
4. Treatment strategies in a patient with concomitant AOID, IgG4-RD and disseminated MAC infection.

## Case presentation

A 61-year-old man of Taiwanese descent was referred to our hospital for evaluation of a new skin rash. One year prior to our evaluation, the patient presented to an outside community hospital with several weeks of constitutional symptoms including fevers, chills and weight loss and was found to have diffuse lymphadenopathy involving his mediastinal, hilum, and axillary lymph nodes. He underwent bronchoscopy with bronchoalveolar lavage which was positive for pulmonary MAC. He was started on treatment with clarithromycin, ethambutol, and rifampin.

**Fig. 1:**
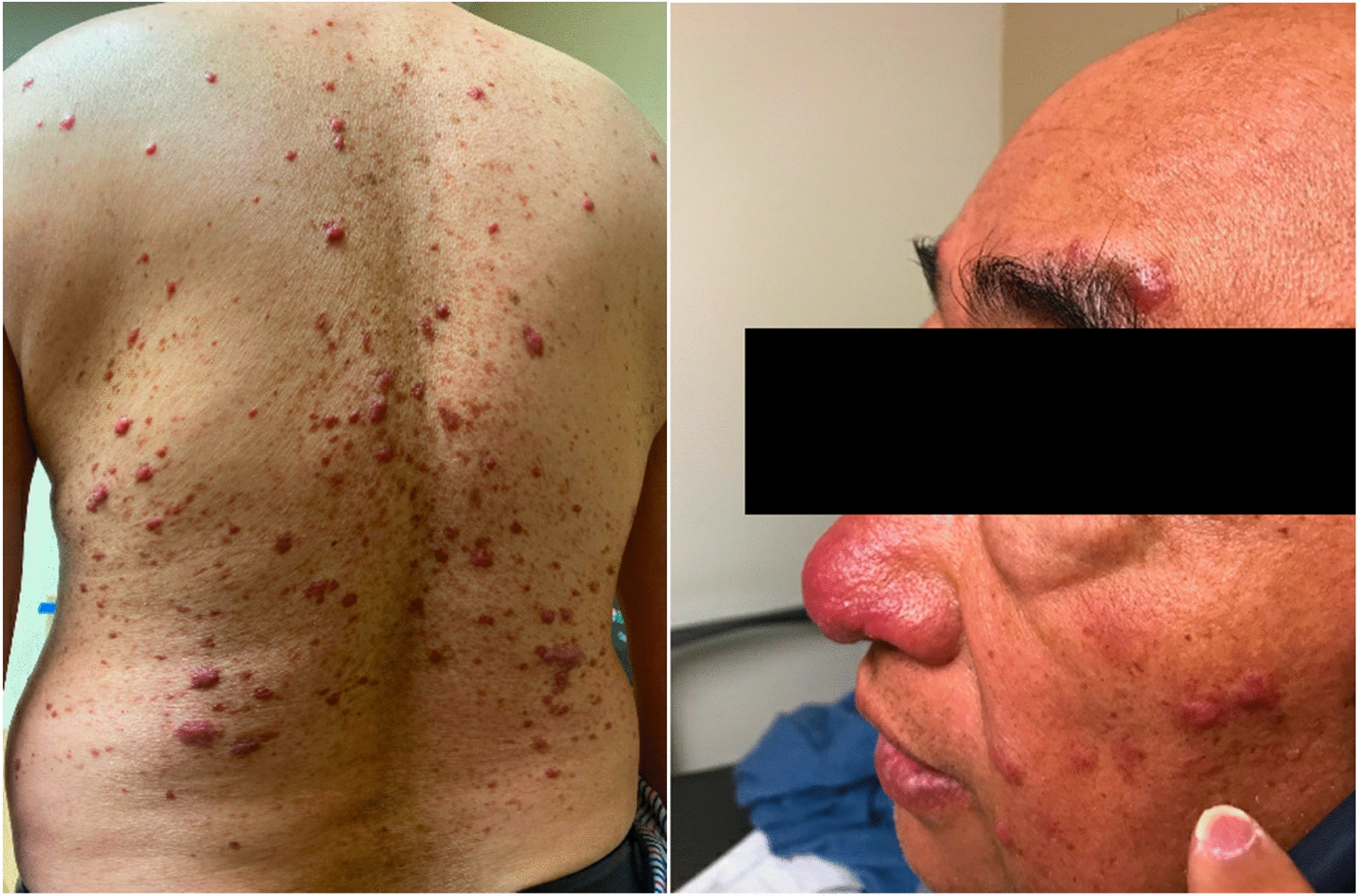
Diffuse maculopapular rash involving face and trunk 3–6 mm in size

Three months into treatment, the patient self-discontinued his ethambutol as he developed a new skin rash that he attributed as a side effect to this antibiotic. The patient was referred to our dermatology clinic at Keck Medicine of University of Southern California (USC) for evaluation. The physical exam was pertinent for a painful diffuse maculopapular rash involving the face and trunk (Fig. 1). Punch biopsies of his skin on his upper back revealed mixed granulomatous and neutrophilic lymphocytic infiltration with plasma cells with extensive confluent sheets of histiocytes and focal emperipolesis suggestive of Rosai-Dorfman disease (RDD).

The patient was also seen in our ophthalmology clinic for bilateral anterior uveitis, which was thought to be secondary to an inflammatory response to his MAC infection vs RDD. The patient was seen in the pulmonary clinic and repeat sputum cultures were negative for MAC and computed tomography (CT) imaging showed stable lymphadenopathy. Due to the reported skin rash side effect, the patient continued dual therapy with clarithromycin and rifampin and was monitored with monthly sputum samples.

Unfortunately, the patient was lost to follow up due to the COVID-19 pandemic. Seven months into treatment of his MAC infection, the patient re-presented to another outside community hospital with worsening constitutional symptoms and weakness to the point at which he was not able to get out of bed. He had a rising leukocytosis to 50 k/mcL with lymphocytic predominance (from a baseline of 10 k/mcL) and progression of his lymphadenopathy for which a core biopsy of his axillary lymph node was performed. Biopsy did not reveal histopathological findings of RDD—it revealed lymphocytic infiltration with IgG4+plasma cells up to 70 per high power field, but with an IgG4+/IgG ratio of < 30%. This did not meet criteria for a diagnosis of IgG4-RD. Acid fast bacilli (AFB) stain was negative. Flow cytometry and molecular NeoGenomics tests came back negative. The patient was referred to our hematologist who recommended an excisional lymph node biopsy, a bone marrow biopsy, and a positive emission tomography (PET) scan.

**Fig. 2:**
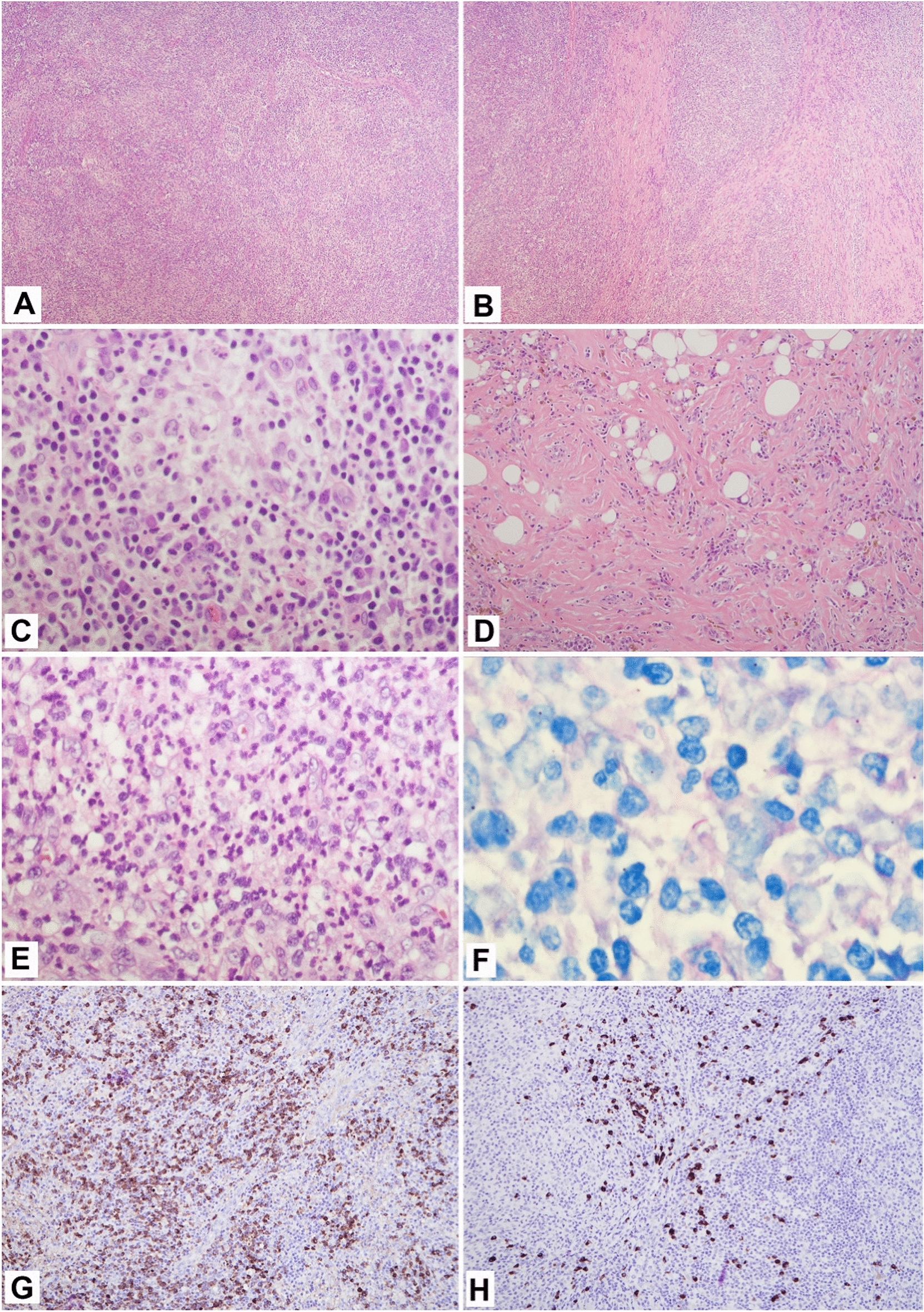
Excisional biopsy of right axillary lymph node; **A**, Effacement of normal lymph node architecture by mixed inflammatory cells (H, E × 40); **B**, Parenchymal fibrosis (H, E × 40); **C**, Collection of histiocytes (H, E × 400); **D**, Capsular fibrosclerosis with inflammatory cells distributed in a storiform pattern (**H**, **E** × 400); **E**, Microabscess formation (H, E × 400); **F**, Positive for acid fast bacilli (AFB stain × 1000); **G**, IgG-positive plasma cells (immunohistochemical stain, × 100); **H**, IgG4-positive plasma cells focally up to 130 per high power field, IgG to IgG4 ratio about 30–40% (immunohistochemical stain × 100)

The PET scan revealed diffuse hypermetabolic lymphadenopathy involving his supraclavicular, mediastinum, hilar, and axillary lymph nodes. Thus, a repeat excisional biopsy of the right axillary lymph node was performed showing a markedly expanded population of mixed inflammatory cells replacing most of the lymph node architecture, including neutrophils, small lymphocytes, plasma cells, and scattered immunoblasts (Fig. 2A). Capsular and parenchymal fibrosclerosis was also present (Fig. 2B, D). Collections of histiocytes and microabscess formation were observed (Fig. 2C, E). AFB special stain was originally interpreted as negative by the pathologist. However, on retrospective, careful re-review by a second pathologist, the stain highlighted rare acid-fast bacilli (Fig. 2F). IgG4+plasma cells were variable in number, from 10 to 120 per high power field in a few foci, up to near 130 per hpf in another focus; the IgG4 to IgG ratio was about 30–40% (Fig. 2G, H). He had elevated serum IgA, IgE, and IgG with an IgG4 level of 308 mg/dL. With these lab findings and organ involvement, he was diagnosed with IgG4-RD.

**Fig. 3:**
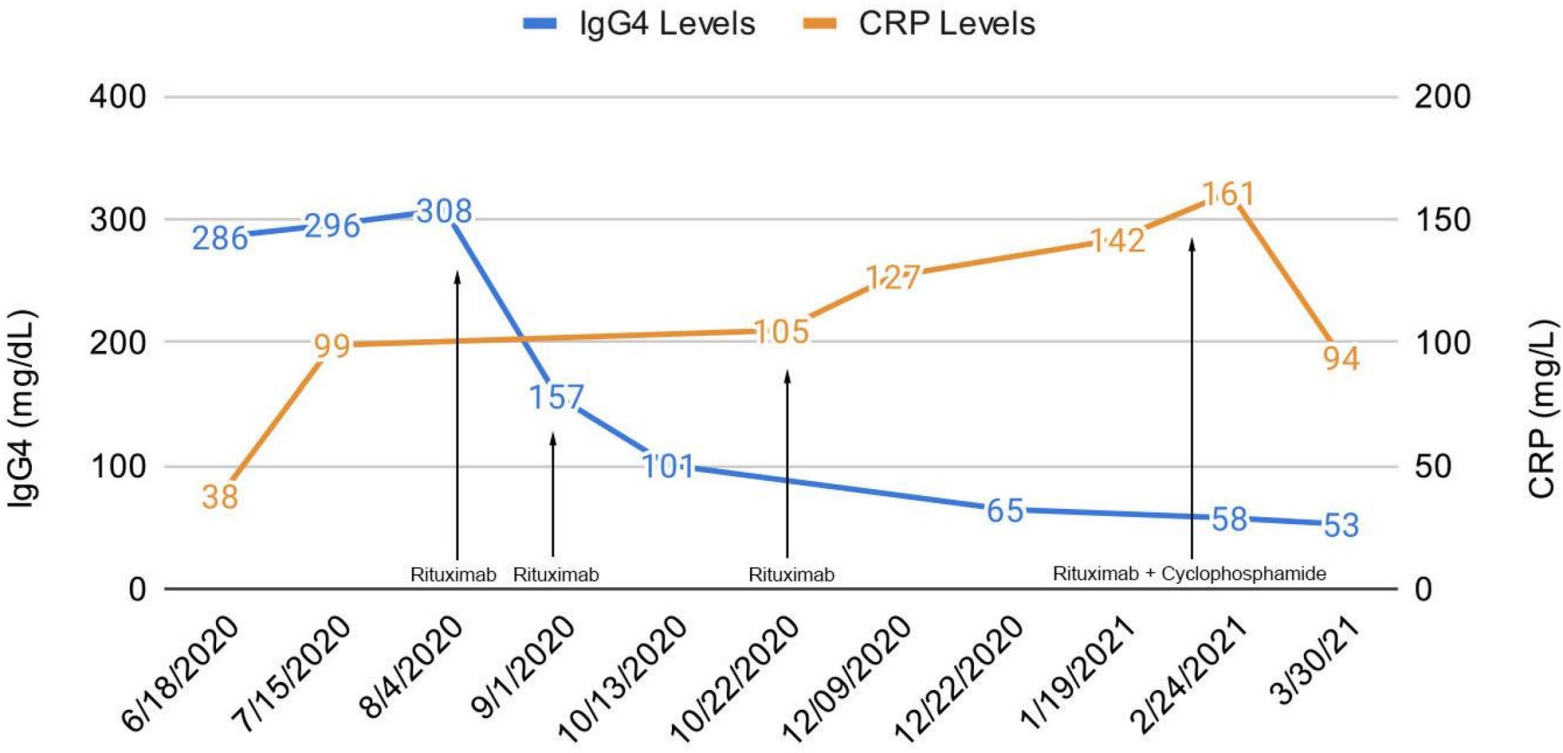
IgG4 levels and CRP levels after initiating treatment for IgG4 disease

A shared decision was made to initiate immunosuppressive therapy for IgG4-RD with dexamethasone 40 mg for 4 days and a rituximab 1000 mg infusion, given the patient had 7 months of negative sputum samples with an initial biopsy interpretation negative for AFB. While his IgG4 levels rapidly responded to treatment, outpatient monitoring of his inflammatory markers were slow to improve despite 3 cycles of the aforementioned immunosuppressive therapy. Eventually 1500 mg of cyclophosphamide was added for the 4th cycle (Fig. 3) with eventual response in both the IgG4 and C-Reactive Protein (CRP) levels.

The patient continued treatment with a 5th cycle of rituximab and a 2nd cycle of cyclophosphamide. Unfortunately, his treatment course was complicated by acute on chronic weakness and back and right knee pain for which the patient presented to the emergency room. He was admitted to the hospital where he was found to have MAC positive septic arthritis of the right knee, MAC positive non-necrotizing granulomas in the bone marrow, and innumerable bony lesions on imaging consistent with a diagnosis of disseminated MAC infection.

The patient was reinitiated on quadruple therapy with amikacin, azithromycin, levofloxacin, and ethambutol for treatment of disseminated MAC infection, and the decision was made to withhold treatment of IgG4-RD pending resolution of his MAC infection. We also confirmed that the patient was adherent to and absorbing his MAC treatment without issues by measuring his serum drug levels of rifabutin, levofloxacin, and azithromycin which came back normal to high. Finally, we confirmed that his second MAC infection was susceptible to macrolide therapy.

The constellation of symptoms of disseminated MAC infection, neutrophilic dermatitis, lymphadenopathy, and polyclonal hypergammaglobulinemia raised suspicion for an immunodeficiency syndrome. An antibody panel to evaluate anti-IFN-gamma autoantibodies was sent which came back negative. Unfortunately, this was likely a false negative given it had been collected after the patient had already received 7 months of treatment with immunosuppressives for IgG4-RD.

**Fig. 4:**
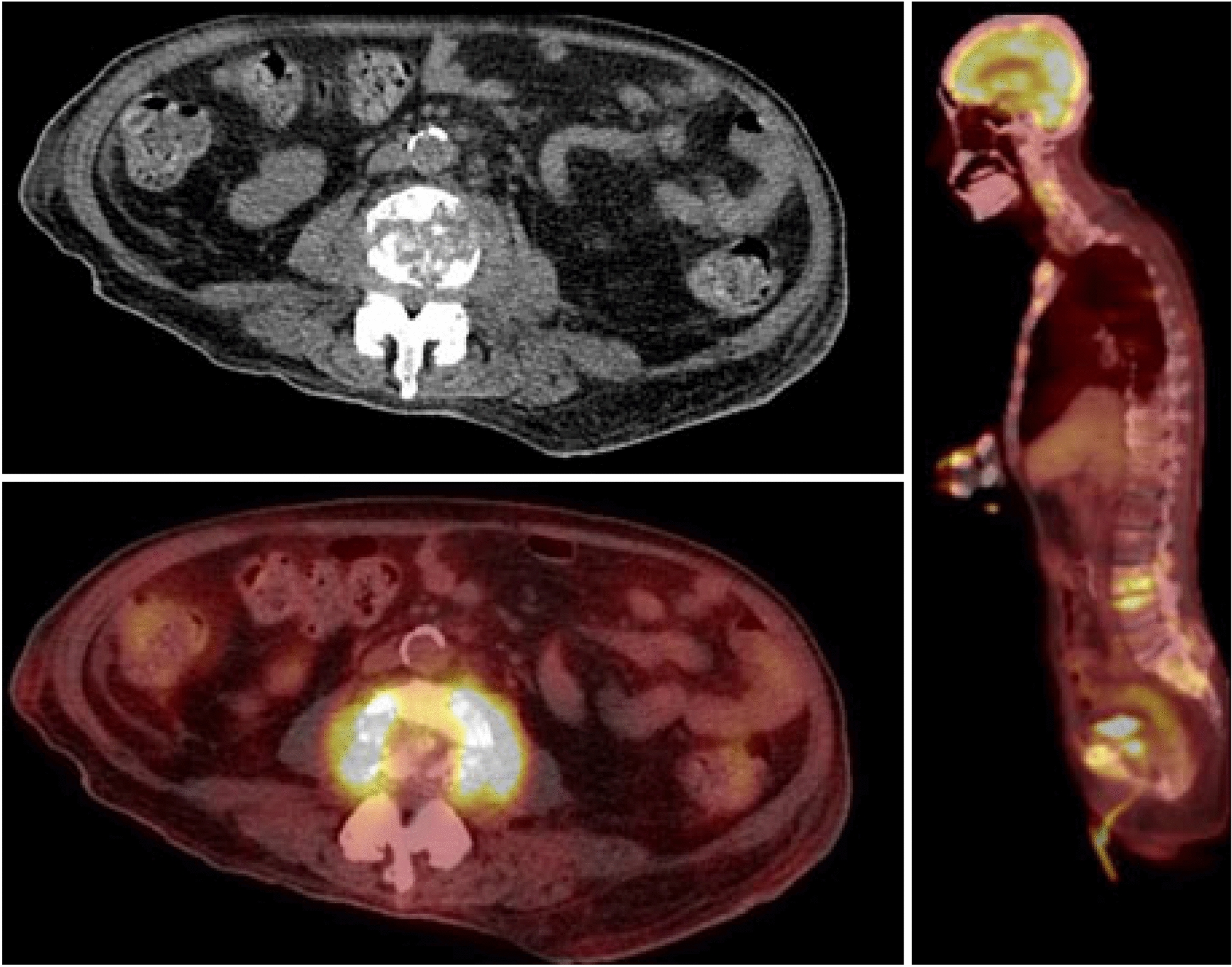
PET/CT scan 10/28/21 Multifocal hypermetabolic mixed lytic and sclerotic lesions were seen throughout the axial and appendicular skeleton. Most significantly, there were pathologic compression fractures of the L3 vertebral body with an associated hypermetabolic soft tissue mass extending into and narrowing the central spinal canal

Despite being on quadruple therapy for disseminated MAC infection, the patient re-presented a third time to the hospital with acute urinary retention and back pain. A PET scan revealed pathologic compression fractures of the L3 vertebral body with an associated phlegmon (Fig. 4). CT guided biopsy returned positive for MAC, suggesting treatment refractory disseminated MAC. Thus, a second IFN-gamma autoantibody panel was sent through the National Institute of Health (NIH) as the patient was no longer on immunosuppressive agents. This came back strongly positive at greater than 22,000. The patient was diagnosed with anti-interferon-gamma autoantibody-associated immunodeficiency syndrome, and the decision was made to continue antibiotic treatment for a 4 year course. Unfortunately, the patient continued to decline, requiring repeated hospitalizations, and the decision was ultimately made to pursue hospice care.

## Discussion

Differential diagnosis for skin rash with nodal involvement is broad including malignancy, disseminated MAC, and autoimmune process. The presence of enlarged histiocytes with foci of emperipolesis, or the engulfing of mature immune cells by histiocytes with abundant cytoplasm, initially raised the suspicion for a potential diagnosis of Rosai-dorfman disease [8]. However, typically in RDD immunohistochemical staining also shows expression of CD68 and S100, but not CD1a which we did not find in our patient [8]. There have also been several case reports of RDD diseases mimics with Rosai-Dorfman like histological features including non-tuberculosis mycobacterial infections [9]. Our patient’s skin biopsy was negative for acid fast bacteria making MAC infection less likely.

**Fig. 5:**
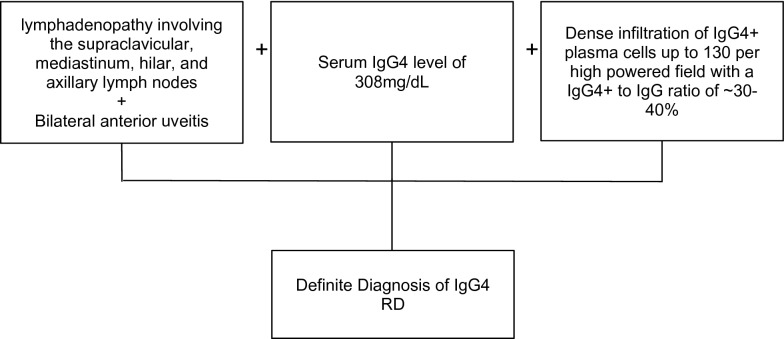
Diagnostic algorithm for IgG4-RD

Rosai-Dorfman-like histological features can also be seen in IgG4-RD [10]. The discovery of hypergammaglobulinemia with elevated IgG4, characteristic histopathological findings and multi-organ involvement led to the diagnosis of IgG4-RD. Diagnostic criteria IgG4-RD was first established in 2011 by Umehara et al. (Table 1, Fig. 5) and later updated in 2020 to exclude solitary nodal disease as criteria for diagnosis [11]. Our patient met all 3 of the diagnostic criteria, with his uveitis thought to represent multiorgan involvement, making a definite diagnosis of IgG4-RD.

**Table 1:** Consensus diagnostic criteria for IgG4-RD

IgG4 related disease diagnostic criteria	
Criteria I	Organ enlargement, mass or nodal disease, or organ dysfunction
Criteria II	A serum IgG4 concentration of > 135 mg/dL
Criteria III	Histopathological findings characteristic of IgG4-RD: IgG4+/IgG+cell ratio> 40% and > 10 IgG4+plasma cells per high-power field

Igg4 related disease diagnostic algorithm	
Diagnostic criteria met	Diagnosis probability
Criteria I, II, III	Definite
Criteria I, III	Probable
Criteria I, II	Possible
No criteria met	Negative

Major histopathologic features for extranodal IgG4-RD have been reported with increased specificity for IgG4-RD. These include a characteristic storiform pattern of fibrosis seen on histopathology as was seen intranodally with our patient [5, 12, 13]. However, various histologic patterns have been described in IgG4-related lymphadenopathy: Type I, multicentric Castleman disease-like; Type II, follicular hyperplasia; Type III, interfollicular expansion; Type IV, progressive transformation of germinal centers; and Type V, inflammatory pseudotumor-like. These pathologic findings are different than those seen in extranodal disease, with the exception of the type V pattern, and are relatively nonspecific.

Interestingly, a reactive dermatitis with neutrophilic infiltration (commonly seen as Sweet’s syndrome and erythema pustulosis) is also associated with AOID [2, 7, 14]. Our patient’s skin biopsy was positive for neutrophilic infiltration, and this, along with his pulmonary MAC infection, was an early and important diagnostic clue for an underlying diagnosis of AOID. One study found that the positive predictive value for AOID increased from 0.154 in patients with a diagnosis of NTM alone to 1.0 in patients with a diagnosis of NTM and a reactive dermatitis [15]. If seen in the future, this combination of disease should prompt investigation with an IFN-gamma autoantibody assay.

In a Thailand and Taiwan cohort, IFN-gamma auto-antibodies were detectable in over 80% of patients suffering from disseminated NTM without previously known risk factors such as human immunodeficiency virus (HIV) [2]. Diagnosis of our patient was eventually established by the presence of anti-IFN-gamma autoantibodies in the serum. This diagnosis was delayed, however, due to concomitant treatment of IgG4-RD that resulted in an initial false negative anti-IFN-gamma level. This reinforces the need for high clinical suspicion of immunodeficiency in a patient of Southeastern Asian descent with reactive dermatitis and an opportunistic infection without known risk factors. If there is such a suspicion, cessation of any immunosuppressive treatment should be considered for prompt diagnosis.

The relationship between IgG4-RD and AOID is an area that requires further research and has only been described twice before to our knowledge [6, 7]. The cause of anti-IFN-gamma autoantibody generation in AOID is unknown, but research suggests there may be a genetic component as there is an association with allele polymorphisms in human leukocyte antigens DRB1 and DQB1 [16]. IFN-gamma regulates the toll-like receptor pathway in response to intracellular pathogens. The disruption of this pathway and subsequent reactive oxygen species production, antigen presentation and interleukin 12 production leads to susceptibility to NTM disease such as MAC [1].

To date, the pathogenesis of IgG4-RD is also incompletely understood. Some have suggested that IgG4-RD is precipitated by a likely unknown antigen that causes B cell differentiation into IgG4 producing plasma cells leading to the increased expression of T helper 2 (Th2) cells and cytokines [17, 18]. The immune mediated response of IgG4-RD shares a similar response to tuberculosis infection as the activation of Helper T cells and the expression of similar cytokines in IgG4-RD have a crucial role in our immune mediated defense against tuberculosis infection [19, 20].

As disseminated mycobacterial disease has been shown to increase a Th2 immune mediated response, it has been suggested that prolonged opportunistic infections due to underlying AOID results in activation of Th2 cells and repeated antigen exposure causes increased IgG4 production [6, 21]. Another possible mechanism of overlap between the two diseases is due to the generation of anti-IFN-gamma autoantibodies itself. It was found that the level of anti-interferon-gamma IgG4 was disproportionately high compared to the total IgG subclass distribution, suggesting that the auto antibodies are excessively of IgG4 subtype [2].

Management strategies of both diseases involve steroids and biologic agents. International consensus guidelines recommend that all symptomatic patients with IgG4-RD and most asymptomatic patients with the potential for irreversible organ damage receive induction therapy due to the metachronus nature of the disease [5]. Induction therapy includes prednisone at a dose range of 30–40 mg daily for at least 2–4 weeks with a following taper that can range from 3 months to 3 years based on expert opinion [5]. Most experts also agree with the addition of a steroid sparing biologic agent to induce more durable remission with less relapse and to minimize toxicities from prolonged steroid exposure [5]. Options for biologic therapy include rituximab, cyclophosphamide, azathioprine, 6-mercaptopurine, mycophenolate mofetil, methotrexate, tacrolimus, and leflunomide.

Treatment for AOID is an area of ongoing research with no published guidelines to guide management choices. Multiple treatments have been trialed in case reports with varying success including intravenous immunoglobulin, subcutaneous IFN-gamma, plasmapheresis, cyclophosphamide and rituximab [3, 22–26]. One study featured a modified lupus nephritis protocol with 5–35 cycles of cyclophosphamide (400 mg) with adjunct steroid use and 10–48 months of antibiotic use and had remission with resolution of infection in 2 out of 7 patients, continued infection without hospitalization in 3 out of 7 patients, and relapsed disease and hospitalization in 2 out of 7 patients [22]. Another study infused rituximab according to a lymphoma regimen (375 mg/m^2^ weekly) for 9–15 cycles over 1–5 years in 4 patients with periods of relapsed disease but overall clinical, radiologic, and laboratory improvement [26]. Early data suggests that management requires both anti-microbial treatment as well as reestablishment of the IFN-gamma pathway through immunosuppressive therapy [26]. This presents a unique, clinical challenge as immunosuppressive treatment can prolong highly morbid infections.

The management of pulmonary MAC in general remains challenging, especially in an immunocompromised host. The advent of macrolide-based regimen was a turning point in MAC treatment, leading to its foundational recommendation in guidelines since 1997 [27–29]. The most recent 2020 guideline recommends a macrolide-based regimen over non-macrolide-based regimen based on extensive observational studies showing higher rates of culture-conversion and that macrolide susceptibility predicts treatment success [27]. It further recommends a three-drug over two-drug regimen to mitigate risk of emerging macrolide resistance and a duration of at least twelve months after culture conversion [27]. But treatment success remains essentially a coin toss with an overall success rate of 52.3% and up to only 61.4% in those adhering to guideline-based therapy [30].

While immunosuppression is an established risk factor for mycobacterial disease, robust evidence-based guidelines are lacking for most immunocompromised hosts, including patients with transplants [31] and those like our patient with a rare immunodeficiency. The best evidence comes from patients with HIV and disseminated MAC. Treatment recommendations essentially remain the same as those for immunocompetent patients, but the guideline recommends primary prophylaxis with a macrolide in those with CD4 counts less than 50 cells/mm3 and, specifically, not taking antiretroviral therapy (ART), replacing the prior recommendation to prescribe prophylaxis to all patients with CD4 counts less than 50 cells/mm^3^ regardless of ART [32]. This change reflects the cornerstone importance of reconstituting immunity as a means to treat and prevent mycobacterial diseases, a strategy we attempted in our patient.

The largest longitudinal study on AOID treatment found that infection clearance was dependent on the use of immunosuppressives [33]. The median time of infection clearance was 3 years with antibiotics alone, 4 years with antibiotics and rituximab, 5 years with antibiotics and cyclophosphamide [33]. We elected to pursue a 4 year course of antibiotic treatment in our patient as he had received 5 cycles of rituximab and 2 cycles of cyclophosphamide for his IgG4-RD treatment. However, by the time of AOID diagnosis, the patient’s disseminated disease was so pervasive that, despite optimal treatment, that patient was transitioned to comfort care.

This case highlights the diagnostic challenge of concomitant AOID, IgG4-RD and disseminated MAC infection, the severity of morbidity and reviews optimal treatment strategies. Because of the novelty and rarity of these diseases, further research is needed to help classify the spectrum of disease between AOID, IgG4-RD and disseminated MAC infection in order to refine treatment strategies in this growing, high-risk population.

## Data Availability

Not applicable.

## Abbreviations

AOID: Adult onset immunodeficiency disease used synonymously with anti-interferon-gamma autoantibody-associated immunodeficiency syndrome
IFN: Interferon
NTM: Nontuberculous mycobacterium
IgG: Immunoglobulin G
IgG4-RD: Immunoglobulin G4-related disease
MAC: Mycobacterium avium complex
USC: University of Southern California
RDD: Rosai-Dorfman disease
CT: Computed tomography
AFB: Acid fast bacilli
CRP: C-Reactive protein
NIH: National institute of health
HIV: Human immunodeficiency virus
Th2: T Helper 2
ART: Antiretroviral therapy
